# The Better Management of Patients with Osteoarthritis Program: Outcomes after evidence-based education and exercise delivered nationwide in Sweden

**DOI:** 10.1371/journal.pone.0222657

**Published:** 2019-09-19

**Authors:** Thérése Jönsson, Frida Eek, Andrea Dell’Isola, Leif E. Dahlberg, Eva Ekvall Hansson

**Affiliations:** 1 Department of Clinical Sciences Lund, Ortopedics, Lund University, Lund, Sweden; 2 Department of Health Sciences, Division of Physiotherapy, Lund University, Lund, Sweden; University of Tasmania, AUSTRALIA

## Abstract

We evaluated a structured education- and exercise-based self-management program for patients with knee or hip osteoarthritis (OA), using a registry-based study of data from 44,634 patients taken from the Swedish “Better Management of Patients with Osteoarthritis” registry. Outcome measures included a numeric rating scale (NRS), EuroQol five dimension scale (EQ-5D), Arthritis self-efficacy scale (ASES-pain and ASES-other symptoms), pain frequency, any use of OA medication, desire for surgery, fear–avoidance behavior, physical activity, and sick leave were reported at baseline, 3 and 12 month. Changes in scale variables were analyzed using general linear models for repeated measures and changes in binary variables by McNamara’s test. All analyses were stratified by joint. At the 3-month follow-up, patients with knee (n = 30686) and hip (n = 13948) OA reported significant improvements in the NRS-pain, the EQ-5D index, the ASES-other symptoms, and ASES-pain scores with standardized effect size (ES) ranges for patients with knee OA of 0.25–0.57 and hip OA of 0.15–0.39. Significantly fewer patients reported pain more than once weekly, took OA medication, desired surgery, showed fear–avoidance behavior, and were physically inactive. At the 12-month follow-up, patients with knee (n = 21647) and hip (n = 8898) OA reported significant improvements in NRS-pain, EQ-5D index, and a decrease in ASES-other symptoms and ASES-pain scores with an ES for patients with knee OA of –0.04 to 0.43 and hip OA of –0.18 to 0.22. Significantly fewer patients reported daily pain, desired surgery (for hip OA), reported fear–avoidance behavior, and reported sick leave. Following these interventions, patients with knee and hip OA experienced significant reductions in symptoms and decreased willingness to undergo surgery, while using less OA medication and taking less sick leave. The results indicate that offering this program as the first-line treatment for OA patients may reduce the burden of this disease.

## Introduction

Osteoarthritis (OA) of the knee and hip is estimated as the 12th highest contributor to global disability [1]. It is the fastest growing cause of disability worldwide driven by an increasingly older population, and the growing incidence of obesity- and sports-related injuries [2, 3]. First line treatment for people with OA is education, exercise, and weight loss (if necessary) while total joint replacement should only be considered when first line treatment no longer give satisfactory results [4–6]. Unfortunately, there is a discrepancy between recommended treatment and what patients receive. Less than 50% of people with OA seeking care receives the first line management [7, 8].

A previous published systematic review have divided the barriers to the limited uptake of guidelines in clinical practice in four different themes; 1) “OA is not that serious” reflecting a belief that OA is a part of the normal aging, 2) Clinicians perceive they are underprepared to give health care according to guidelines, 3) “Personal beliefs at odds with providing recommended practice” and 4) “Dissonant patient expectations” [9].

Adding to this, recent studies indicate a significant proportion of total knee joint replacement were judged to be inappropriate, suggestion that the clinical implementation of those recommendations may be suboptimal [10, 11]. In patients who received total hip joint replacement in Sweden only 41% have got structured education and exercise prior to the operation [12].

To overcome the discrepancy between guidelines and practice, a Swedish national program, “the Better Management of Patients with Osteoarthritis” (BOA), was initiated in 2008. The BOA program has three branches: 1) education of physiotherapists (PT) and occupational therapists to deliver health care according to guidelines; 2) education of patients through a self-management program; and 3) collection of patient-reported and PT-reported outcomes before and after treatment at the National Quality Register, the BOA registry [13]. The implementation of the BOA program in Sweden has been successful and the program is delivered at a primary care level by more than 700 care units in Sweden [14]. Since the introduction of BOA in Sweden, similar self-management programs have successfully been initiated and implemented in Denmark and Norway, through the GLA:D® [15] and AktivA programs [16], respectively. Also a digital self-management program based on BOA has been developed in Sweden [17].

Previous studies and systematic reviews have shown that self-management programs with education and exercise for patients with knee and hip OA can lead to decreased pain [15, 18] increased health-related quality of life (QoL) [15, 19, 20], increased self-efficacy [18], decreased willingness of surgery [21] and delayed surgery with knee or hip replacement [22, 23]. Unfortunately, multicenter studies evaluating the results from these interventions when implemented in the real-word setting are scares.

The specific aims of BOA are; 1) decreased pain, 2) improved QoL, 3) increased ability to self-manage the disease, 4) reduce number of patients taking OA medication, 5) decreased fear–avoidance behavior, 6) increased level of physical activity, 7) decreased willingness of surgery and 8) reduce the amount of sick leave [14].

The purpose of this study is to evaluate the changes in outcomes related to the specific aims of the BOA program in patients with knee and hip OA after participation in the program.

## Materials and methods

This was an observational registry-based study evaluating changes in outcome variables in patients following BOA program, including patients with data available at baseline and at 3- or 12-month follow-ups between 2008 and 2016. The BOA registry contains patient-reported outcomes from participants in a self-management program including education and exercise for patients with knee and hip OA.

For inclusion in this study, the worst joint defined by the PT should be the knee or hip, and the participant should have taken part in at least the theory sessions. A 2-month delay for the 3-month follow-up and a 3-month delay for the 12-month follow-up were allowed. All analyses were stratified by the most affected joint, knee or hip. The study was approved by the Regional Ethical Review Board in Gothenburg (1059–16). All patients received oral and written information about their registration in the BOA registry.

### BOA program

The BOA program was implemented in Sweden in 2008 and had three branches: 1) education of PTs and occupational therapists; 2) education of patients; and 3) collection of patient-reported and PT-reported outcomes before and after treatment at the National Quality Register, the BOA registry [13] (Tables 1 and 2).

**Table 1:** Patient-reported outcomes in the BOA registry.

Variable	Baseline	3-month follow-up	12-month follow-up
Age	x	x	x
Gender	x	x	x
Born in Sweden	x		
Swedish citizen	x		
Educational level	x		
Current employment	x		
Sick leave	x		x
Smoking	x		
Weight (kg)	x		x
Height (cm)	x		x
Body mass index (kg.m^2^)	x		x
Most affected joint: knee/hip/hand/shoulder	x	x	x
Other affected joints: hip/knee/foot/hand/elbow/ shoulder/back/neck	x	x	x
During the last week to what degree have ailments originating from your arm, shoulder or hand impaired your work or other daily activities?	x	x	x
Mean pain intensity during the last week in the most affected joint	x	x	x
Fear–avoidance behavior	x	x	x
Willingness to undergo surgery of knee/hip	x	x	x
EuroQol-5 dimensions	x	x	x
EuroQol Visual analogue scale	x	x	x
Minutes per week of physical activity	x	x	x
Personal level of physical activity compared with others of the same age	x	x	x
Arthritis Self-Efficacy Scale (subscales: pain and other symptoms)	x	x	x
Satisfaction with the BOA program	x	x	x
Frequency of using what was learnt in the BOA program	x	x	x

**Table 2:** Physiotherapist-reported outcomes in the BOA registry.

Variable	Baseline	3-month follow-up
Duration of symptoms	x	
Most effected knee/hip/hand joint	x	x
Other affected knee and hip joints	x	x
Prior surgery of most affected joint	x	
Prior surgery to contralateral side	x	
X-ray of most affected joint	x	x
MRI of most affected joint	x	x
On waiting list for surgery	x	x
Prior explanation of knee/hip problems	x	
Prior treatment of knee/hip problems	x	
Prior information about weight reduction	x	
Use of painkillers and type	x	x
Using walking aids	x	
Treatment other than the BOA program during follow-up		x
Participation in theory sessions and number of supervised exercise sessions in the BOA program		x

MRI, magnetic resonance imaging.

### Education for PTs and occupational therapists

Between 2008 and 2016, around 3000 PTs and occupational therapists interested in the BOA program participated in a 1- or 2-day training course showing how to diagnose OA and deliver OA care as described in the clinical guidelines. In addition, they received access to digital material, including PowerPoint^®^ presentations, to support them in the delivery of the BOA program and to maximize the adherence to the program.

### Education and exercise for patients

**Fig 1:**
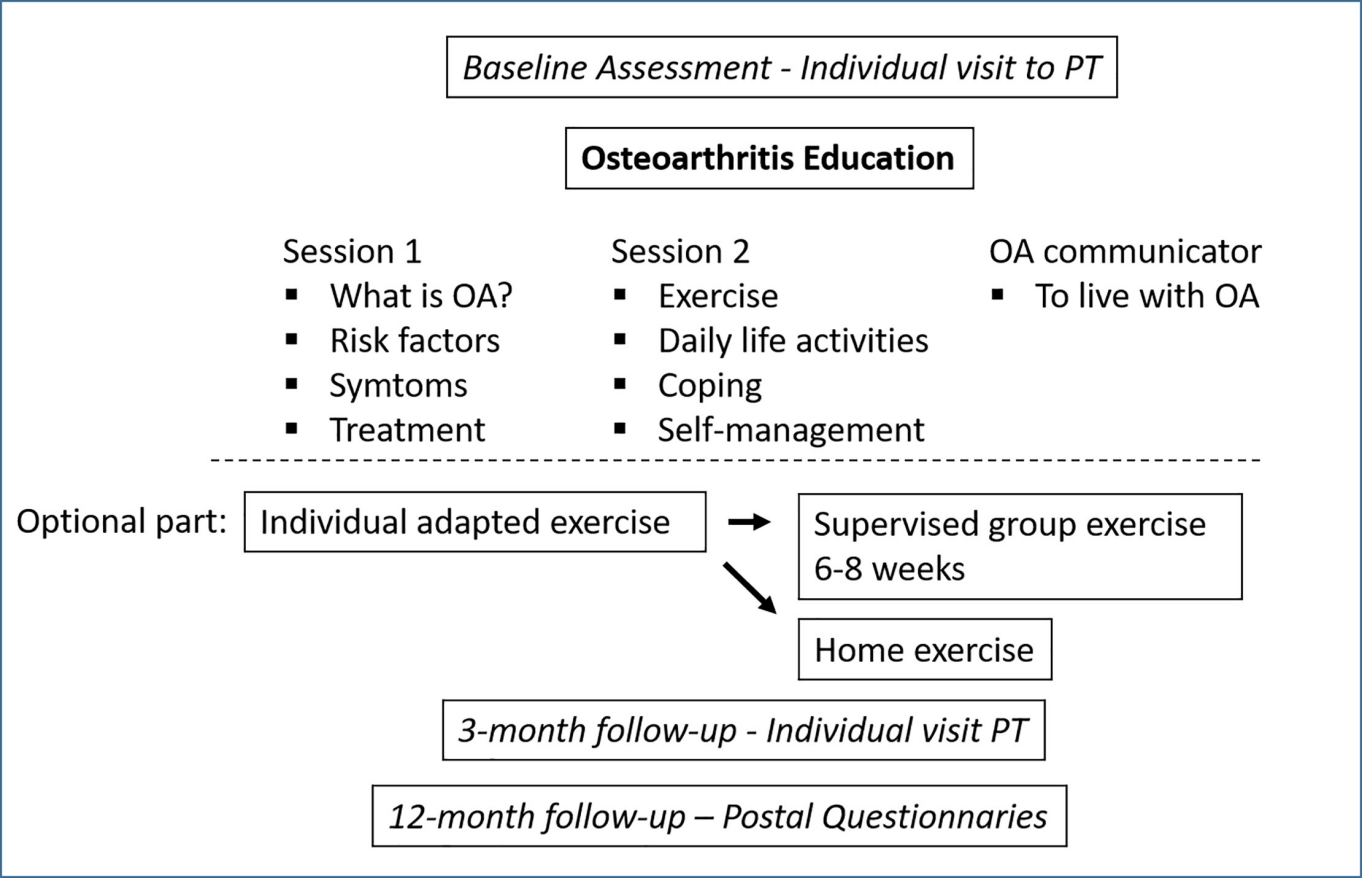
Concept of the education and supervised exercise program according to the BOA program. PT, physiotherapist.

The BOA program for patients have been described elsewhere [13], (Fig 1). Briefly, they consist of a minimum of two theoretical group sessions of about 90 min lead by a PT with 7–12 participants in each group (Fig 1). The minimal intervention could not be modified, but each PT could choose to adjust the content above and beyond the minimal intervention to suit their clinical routines and resources. In a one-to-one session, the patients were introduced to the individual exercise program by a trained PT, based on their specific needs and goals. Patients could thereafter choose to perform the exercises on their own (at home), or during PT-supervised group exercise classes twice a week for 6 weeks. To support an active lifestyle, an individual visit was scheduled 3 months after the first visit, regardless of whether patients chose to participate in the supervised group exercise. Patients were also informed that a 12-month follow-up would be conducted, based on a postal questionnaire.

### Patients

The program is delivered at a primary care level by more than 700 care units in Sweden. Health care is primarily financed through public taxes in Sweden, with a maximum fee of approximately 120 USD for outpatient’s visits during a twelve month period aiming to minimize the financed barriers for seeking health care.” Therefore, patients can choose to attend at private or public health for the same maximum fee.

The inclusion criteria for patients to access the BOA program were diagnoses of symptoms from knee and/or hip OA that resulted in contact with the health care system. The exclusion criteria were: a reason other than OA for joint problems (e.g., sequel hip fractures, chronic widespread pain; inflammatory joint diseases or cancer); total joint replacement within the past 12 months; other surgery of the knee or hip joint within the past 3 months; and patients not able to read or understand Swedish. Radiographs are not needed to diagnose OA according to international guidelines [24] and were therefore not part of the eligibility criteria.

### Outcome measures

A questionnaire was completed by the PT at baseline and at 3-month follow-up (Table 2) and a questionnaire were completed by the patients at baseline, 3 and 12-month follow-up (Table 1). At baseline and at the 3-month follow-up the questionnaire was filled in at the clinic and the treated PT did input the data to the registry. At 12-month follow-up the questionnaire was sent by post to the patient with a prepaid envelope to send it back to the BOA registry and personal from the registry did input the data. Comorbidity were measured by the Charnley score, categorizes patients into one of three groups: A—one joint with OA (unilateral knee or hip); B—bilateral OA (both knees or both hips); C—OA in multiple joint sites (hip and knee), or presence of any other disease that affects walking ability [25].

#### Pain

Mean pain intensity during the last week in the most affected joint was evaluated on a numerical rating scale (NRS) of 0–10 [26, 27]. Pain frequency was assessed by the question: “How often do you have pain in your knee/hip” with five possible answers: never, every month, every week, every day, or all the time. For the purpose of this study to get an overview of the amount of patient who change from daily pain to weekly or less pain, we dichotomized this question into, frequent pain (every day, or all the time) and rare pain (never, every month or every week,).

#### Quality of life

QoL was assessed using the EuroQol five-dimensional (EQ-5D) tool, which has been used previously for measuring the outcomes of interventions in patients with OA [19, 28]. EQ-5D can be presented as a health profile or as a global health index with a weighted total value (here the UK tariff was used), where the minimum value is –0.594 and the maximum is +1.0 [29]. For the purpose of this study, we used the EQ-5D index to assess overall QoL.

#### Ability to self-manage the disease

The ability to self-manage the disease was assessed by the Arthritis Self-Efficacy Scale (ASES), which is designed to measure confidence in one’s own ability to manage chronic arthritis. It consists of 20 statements, divided into three subscales: pain, function, and other symptoms. Each item is scored on a 10-point Likert scale ranging from 10 (very uncertain) to 100 (very certain) [30]. This study used only the subscales for pain and other symptoms. ASES has been used previously to evaluate patient education programs for patients with arthritis [30, 31]. The Swedish version has been proven valid [32].

#### Intake of OA medication

Intake of medication was evaluated by the PT asking the patients whether they had taken any OA medication including painkillers during the last 3 months because of their knee/hip pain. (Yes/No).

#### Fear–avoidance behavior

Fear–avoidance behavior was assessed by the question: “Are you afraid your joints will be injured by physical training/activity?” (Yes/No).

#### Physical activity

Physical activity was assessed by the question: “How many days are you physically active for at least 30 minutes a day during a regular, typical week?” There were eight possible answers: 0, 1, 2, 3, 4, 5, 6, or 7 days. This question was developed by the National Board of Health and Welfare in Sweden for population-based studies on physical activity and has been validated against an accelerometer [33]. For this study, the question was dichotomized into <150 or ≥150 min/week based on recommendations for physical activity [34].

#### Willingness to undergo surgery

Willingness to undergo surgery was assessed by the question: “Are your knee/hip symptoms so severe that you wish to undergo surgery?” (Yes/No).

#### Sick leave

Sick leave was assessed by the question: “What does your work situation look like today? Tick the option that best suits your situation.” There were five possible answers: 1) working or in full-time study; 2) on full-time sick leave; 3) on part-time sick leave (defined as being on sick leave on part of the work time, but not full time); 4) retired; and 5) unemployed. For the purpose of the study, we dichotomized this question to be on full- or part-time sick leave as “yes” or “no”.

#### Statistical analyses

All analyses were performed using IBM SPSS Statistics (Version 25, IBM Corporation, Armonk, NY, USA). The significance level was set at P < 0.05. The changes in scale variables (NRS pain, EQ-5D, ASES-other symptoms, and ASES pain) between baseline and 3 months, and between baseline and 12 months were analyzed using general linear models (GLMs) with repeated measures. The results were presented as means with 95% confidence intervals (CIs). Standardized effect sizes (ESs) for scale variables were presented as Cohen’s *d*: (x–x/S_average_). ES were considered as small (d = 0.2), medium (d = 0.5) or large (d = 0.8) [35]. Changes in binary variables (pain frequency, intake of OA medication, willingness to undergo surgery, fear–avoidance behavior, physical activity, and sick leave) were analyzed using McNemar’s test.

## Results

### Subjects

Baseline characteristics of the 72,131 patients with OA were included in the BOA registry from May 1, 2008 to December 31, 2016 are presented in Table 3. Of these, 44,634 attended the 3-month follow-up and 30,545 attended the 12-month follow-up. All individuals included in the intervention group attended the theory component, and 86% of those with knee or hip OA also received an individual adapted exercise program. Of these, 30% participated in supervised exercise 10–12 times, 12% participated 7–9 times, 17% participated 1–6 times, and 41% did not participate in supervised exercise.

**Table 3:** Baseline characteristics in the Better Management of Patients with Osteoarthritis (BOA) program.

	Knee OA			Hip OA		
Variable	Total	Excluded at 3-month follow-up (n = 18,729)	Excluded at 12 month follow-up (n = 27,768)	Total	Excluded at 3-month follow-up (n = 8768)	Excluded at 12-month follow-up (n = 13,818)
	(n = 49,415)			(n = 22,716)		
Women, % (n)	69 (33,954)	68 (12,508)	67 (18,660)	68 (15,358)	66 (5,808)	66 (9,088)
Age, mean (± SD; range) in years	66 (10; 18–97)	65 (10; 19–97)	65 (10; 18–95)	67 (10; 27–94)	66 (10; 28–84)	66 (10; 28–94)
Body mass index in kg/m^2^, mean (± SD; range)	28.5 (4.9; 15.1–59.9)	28.7 (5.2; 15.1–59.9)	28.7 (5.2:15.1–59.9)	27.1 (4.4; 15.2–59.6)	27.3 (4.9; 16–59.5)	27.3 (4.7; 15.2–59.6)
Smoker, yes; % (n)	7 (3,385)	8 (1,506)	8 (2,217)	8 (1,819)	9 (810)	9 (1,213)
Charnley Category*, %						
A	38	38	39	38	38	38
B	23	22	24	10	10	10
C	39	39	37	52	52	52
Education level, %						
Elementary school	34	34	35	35	35	35
High school	38	39	39	36	36	37
University	28	27	26	29	29	28
Willingness to undergo surgery, % (n)	25 (12,214)	29 (5,183)	29 (7,980)	30 (6,860)	33 (2,920)	37 (5,066)
Fear–avoidance behavior, % (n)	18 (8,897)	19 (3,595)	19 (5,372)	15 (3,390)	16 (1,375)	16 (2,212)
Pain intensity during the last week, NRS (0–10), mean (SD)	5.3 (2)	5.5 (2)	5.5 (2)	5.5 (2)	5.7 (2)	5.7 (2)
Pain frequency Daily pain or all the time % (n)	82 (40,195)	81 (15,246)	82 (22,798)	84 (19,144)	86 (7,426)	87 (11,932)
Patients’ intake of OA medication over the last 3 months, % (n)	75 (37,045)	75 (14,020)	76 (20,977)	77 (17,387)	76 (6,701)	79 (10,812)
Patients on sick leave during the last 12 months, % (n)	14 (7,057)	17 (3,095)	17 (4,633)	20 (4,436)	23 (2,012)	22 (3,013)
EQ-5D**, mean score (± SD)	0.625 (0.230)	0.606 (0.245)	0.604 (0.244)	0.596 (0.243)	0.571 (0.257)	0.567 (0.257)
Arthritis self-efficacy scale pain (10–100), mean (± SD)	63 (19)	62 (20)	62 (19)	60 (19)	58 (20)	58 (20)
Arthritis self-efficacy scale other symptoms (10–100), mean (±SD)	67 (17)	66 (18)	66 (18)	65 (17)	64 (18)	64 (18)
Physically inactive, % (n) ***	51 (24,993)	50 (9,458)	53 (14,732)	51 (11,579)	53 (4,630)	53 (7,260)

* Charnley Category A, one joint with OA (unilateral knee or hip); B, bilateral OA (both knees or both hips); C, OA in multiple joint sites (hip and knee), or presence of any other disease that affects walking ability

** EQ-5D, EuroQol five-dimensional

***physically inactive was defined as <150 min per week at moderate intensity.

### Excluded from analysis at the 3-month and 12-month follow-up

**Fig 2:**
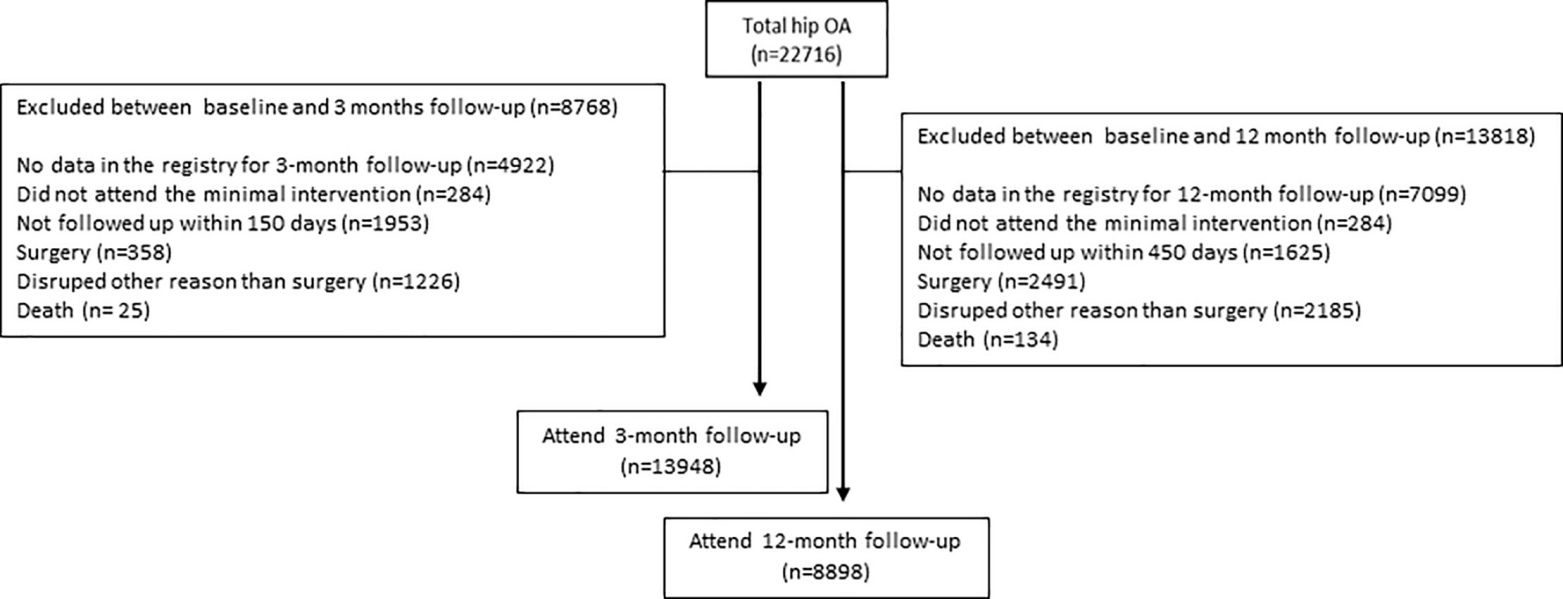
Flowchart of the study, knee OA.

**Fig 3:**
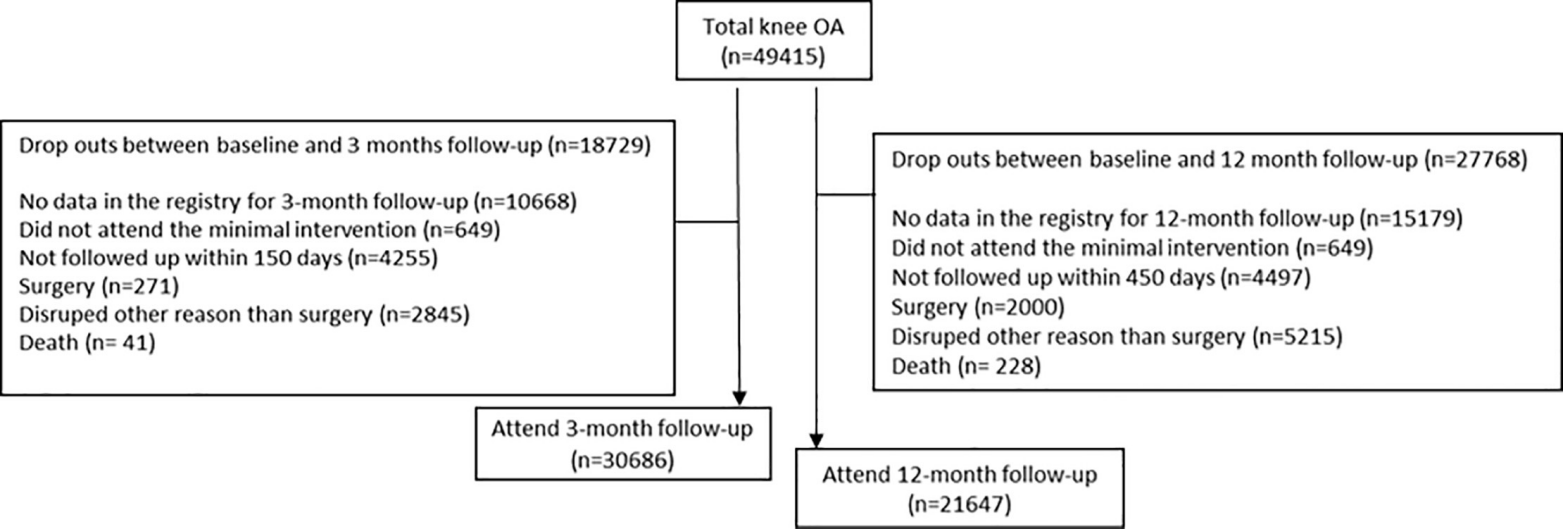
Flowchart of the study, hip OA.

Data from 39% of the patients were excluded at 3-month follow-up and 50% of the patients at the 12 month follow-up (Figs 2 and 3). Baseline characteristics of the participants excluded at 3 and 12 month, for each joint, are listed in Table 3.

### Results at the 3-month follow-up

Between baseline and the 3-month follow-up, patients with knee and hip OA showed statistically significant improvements in scores for NRS-pain, the EQ-5D index, ASES-other symptoms, and ASES-pain tools. Standardised ES (Cohen’s *d*) was 0.25–0.57 for patients with knee OA and 0.15–0.39 for those with hip OA (Table 4). Furthermore, statistically significantly fewer patients with knee and hip OA reported daily pain, took OA medication, had willingness to undergo surgery, reported fear–avoidance behavior, and were physically inactive (Table 5).

**Table 4:** Mean (SD) differences in numeric rating scale (NRS)/pain, EuroQol five-dimensional (EQ-5D-index), Arthritis Self-efficacy Scale (ASES)/pain, and Arthritis Self-efficacy Scale (ASES)/other symptoms, between baseline and 3-month follow-up and between baseline and 12-month follow-up.

Outcome	Knee OA									
	*n*	Baseline mean (SD)	3 months mean (SD)	Mean difference (CI)	Cohen’s *d*	n	Baseline mean (SD)	12 months mean (SD)	Mean difference (CI)	Cohen’s *d*
**NRS/pain**	*30*,*501*	5.24 (1.96)	4.07 (2.17)	1.18 (1.15–1.20)	0.57	*21*,*283*	5.15 (1.94)	4.23 (2.32)	0.92 (0.89–0.95)	0.43
**EQ-5D**	*29*,*865*	0.636 (0.220)	0.702 (0.197)	0.065 (0.063–0.068)	0.31	*20*,*571*	0.653 (0.207)	0.692 (0.198)	0.039 (0.036–0.042)	0.19
**ASES/other symptoms**	*27*,*290*	68.1 (16.8)	72.2 (17.0)	4.1 (3.9–4.3)	0.24	*15*,*447*	68.9 (16.3)	68.2 (18.3)	–0.75 (–1 to –0.5)	–0.04
**ASES/pain**	*27*,*575*	64.2 (18.6)	68.9 (19.5)	4.7 (4.5–4.9)	0.25	*15*,*619*	65.1 (18.2)	64.2 (20.6)	–0.9 (–1.2 to –0.62)	–0.04
	**Hip OA**									
**NRS/pain**	*13*,*864*	5.39 (1.93)	4.57 (2.21)	0.82 (0.79–0.85)	0.39	*8*,*736*	5.16 (1.92)	4.7 (2.3)	0.46 (0.42–0.51)	0.22
**EQ-5D**	*13*,*570*	0.611 (0.232)	0.654 (0.225)	0.043 (0.040–0.047)	0.19	*8*,*448*	0.642 (0.211)	0.650 (0.218)	0.009 (0.004–0.014)	0.04
**ASES/other symptoms**	*12*,*339*	66.0 (17.0)	68.6 (17.8)	2.6 (2.4–2.9)	0.15	*6*,*221*	67.7 (16.4)	64.9 (18.8)	– 2.8 (–3.2 to –2.4)	–0.16
**ASES/pain**	*12*,*477*	60.5 (18.8)	63.5 (20.6)	3,1 (2.7–3.4)	0.16	*6*,*283*	62.3 (18.2)	58.7 (21.2)	–3.6 (–4.1 to –3.1)	–0.18

CI, confidence interval. SD; Standard deviation,). Standardized effect sizes (ESs) were presented as Cohen’s *d*: (x–x/S_average_). ES were considered as small (d = 0.2)

medium (d = 0.5) or large (d = 0.8)

**Table 5:** Percentage changes in patients taking osteoarthritis (OA) medication, pain frequency, exhibiting fear–avoidance behavior, willingness to undergo surgery, and physical activity between baseline and 3-month follow-up and baseline and 12-month follow-up.

Outcome	Knee OA							
	*n*	Yes at baseline n (%)	Yes at 3-months n (%)	P	n	Yes at baseline n (%)	Yes at 12 months n (%)	P
Intake of OA medication (yes/no)	30,392	22,908 (75)	16,858 (55)	**<0.001**	N/A	N/A	N/A	N/A
Pain frequency (frequent pain/rare pain)*	30,017	24,588 (82)	17,931 (60)	**<0.001**	*21*,*181*	17,117 (81)	11,860 (56)	**<0.001**
Fear–avoidance behavior (yes/no)	30,358	5,268 (17)	1,120 (4)	**<0.001**	21,186	3,435 (16)	856 (4)	**<0.001**
Willingness to undergo surgery (yes/no)	29,739	6,914 (23)	3,602 (12)	**<0.001**	20,992	4,076 (19)	2,085 (10)	0.962
Physical inactivity (yes/no)**	25,419	15,152 (60)	9,999 (39)	**<0.001**	17,328	10,125 (58)	6,812 (39)	0.902
Sick leave (yes/no)***	N/A	N/A	N/A	N/A	7,309	1,031 (14)	366 (5)	**<0.001**
	**Hip OA**							
Intake of OA medication (yes/no)	13,811	10644 (77)	8,551 (62)	**<0.001**	N/A	N/A	N/A	N/A
Pain frequency (frequent pain/rare pain)*	13,720	11595 (85)	9,195 (67)	**<0.001**	8,703	7,085 (81)	5,385 (62)	**<0.001**
Fear–avoidance behavior (yes/no)	13,794	2005 (15)	402 (3)	**<0.001**	8,713	1,146 (13)	278 (3)	**<0.001**
Willingness to undergo surgery (yes/no)	13,580	3891 (29)	2,566 (19)	**<0.001**	8,616	1,705 (20)	1,050 (12)	**<0.001**
Physical inactivity (yes/no)**	11,725	6920 (59)	4,629 (39)	**0.041**	7,279	4,264 (59)	2,860 (39)	0.531
Sick leave (yes/no)***	N/A	N/A	N/A	N/A	2,646	320 (12)	143 (5)	**0.017**

*Daily pain or all the time.

**Physically active <150 min/week.

***All retired persons were excluded.

N/A, not available, the significance level was set at P < 0.05

### Results at the 12-month follow-up

Between baseline and the 12-month follow-up, patients with knee and hip OA reported a statistically significant improvement in NRS-pain and in the EQ-5D index, but a statistically significant decrease in ASES-other symptoms and in ASES-pain scores (Table 4). Standardised ES (Cohen’s *d*) were between –0.04 and 0.43 for those with knee OA and –0.18 and 0.22 for those with hip OA. Furthermore, significantly fewer patients reported daily pain, expressed willingness to undergo surgery (only for hip OA), showed fear–avoidance behavior, and were on sick leave (Table 5)

## Discussion

This is the only published study we are aware of assessing a wide range of outcomes in more than 40,000 patients with knee and hip OA who received a structured self-management program including education and exercise in a real-world setting. Our data show that patients who participated in the program showed significantly reduced OA-related symptoms and increased QoL, as well as improved attitudes toward exercise and reduced OA drug intake and sick leave. Our results are in agreement with GLA:D® in Denmark [15] and previous findings from cohort studies evaluating the effect of education and exercise on pain [36, 37], QoL [19, 20], self-efficacy [18] and willingness of surgery [21].

Previous exercise-based interventions have shown smaller ES improvements for those with hip than knee OA [38, 39] which is in line with the results from this study. The BOA intervention showed medium ES values for pain reduction in patients with knee OA at 3-month follow-up but low ES at 12-month follow-up. These observed ESs are better or similar than those achieved with the use of analgesia and nonsteroidal anti-inflammatory drugs (NSAIDs) but without the risk of severe side effects [5]. In patient with hip OA, the BOA intervention produced only small ES values at 3 and 12 months and these values were similar to those achieved from analgesics [5]. Similarly, QoL increased significantly with small ESs in patients with knee OA while patients with hip OA had no effect at both follow-ups. A review evaluating the ESs at 8 weeks in an exercise program on pain, function, and QoL showed medium to large ES values for pain and function, but only small values for QoL [38], which is in line with our results.

At 3-month follow-up, 20% and 15% fewer patients with knee and hip OA respectively reported taking OA medication. Considering the association between the use of paracetamol, NSAIDs and opioids and increased risk of serious side effects and death [40, 41], a decrease in the consumption of OA medication may lead to fewer adverse events in patients undergoing the BOA program. However, OA-medication may also help patients to exercise more, and it is not clear whether the benefits overcome the risks.

Participating in >12 exercise sessions leads to a larger reduction in OA symptoms compared with participating in <12 sessions [42]. In the BOA program, only 60% of the patients participated in the supervised exercise and of those only 30% participated in exercise for 10–12 sessions. We found significantly improved results for all outcomes at the 3-month follow-up, which tended to attenuate at the 12-month follow-up with self-efficacy, physical activity, and willingness to undergo surgery losing statistical significance. Thus, there is a need to implement actions to enhance patients’ compliance with the intervention and the lifestyle changes required to maintain the improvement experienced after the intervention over an extended period. Indeed, previous studies showed how a reduction in the outcome after an intervention can be expected and how the addition of booster sessions might help to maintain the results over time [43].

Fewer patients reported pain frequent pain (every day or all the time), exhibited fear–avoidance behavior, and were on sick leave after 12 months in the program. Considering that patients with knee OA have double the risk of sick leave compared with the general population, offering education and exercise to all patients with OA can have a significant impact on the societal burden of this disease [44]. However, to what extent nationally imposed limitations on sick leave periods have influenced the reported incidence of sick leave is not clear. The reduction in the number of patients on sick leave up to 12 months after the BOA program is important.

### Strengths and limitations of the study

Our study had a number of limitations. First, being an observational registry study there is no control group included and causal relationships could not be established. Second, data from the BOA registry were collected from different clinical settings and differences in treatment delivery and patient management may have influenced the results. Third, we dichotomized pain frequency into “frequent pain” (pain all the time or every day) and “rare pain” (never, every month or every week). This means that reduction in pain frequency from “all the time” to “every day” or “once a week” may have not been captured in the analyses. Fourth, the amount of excluded patients at 3- and 12-month follow-up were high, this is very common in registry-based studies from real word settings [45]. Around 55% of the excluded patients was due to missed data. Due to the lack of information regarding the reason for the data being missing, it is unclear if the patients have dropped out from the intervention or if the PT missed inputting the data into the registry. We do not know if the excluded patients have better or worse results. In fact, it has been shown that even patients improving during the intervention may discontinue the treatment thinking that exercising further is not necessary while others keep exercising because afraid of getting worse [46]. However, the baseline characteristics of the excluded patients, only differed from those included in the study in the willingness of surgery, and pain frequency (Table 3). This might have affected the results positively, since willingness of surgery and frequent pain could indicate more severe OA symptoms which may be harder to treat with satisfactory results. Fifth, despite all the questionnaire and measurement used in BOA, are validated, most of the data are self-reported, thus the results may be affected by patients wanting to “do well” and please healthcare providers by reporting exaggerated improvements. Also, a potential “regression to the mean” effect must be considered in the interpretation, especially given the lack of control group. However, a previous controlled study evaluating the effect of the BOA program compared to a reference group showed similar results to this study for the treatment group, while the control group showed a trend towards worsening in OA symptoms (pain, QoL, self-efficacy) during the study period [18].

The strength of the study is that it included over 40,000 patients who underwent a first-line treatment for OA in different real-world settings in Sweden, thus increasing the generalizability of the results. The results suggest that patients with knee and hip OA who participate in a Swedish self-management program- provided in primary cares show improvements in a variety of symptoms after the program.

## Conclusions

Patients with knee and hip OA who participated in structured education and individually adapted exercises in the BOA program experienced statistically significant reductions in symptoms and decreased willingness to undergo surgery while using less OA medication and taking less sick leave. These results suggest that offering this intervention as the first-line treatment for patients with OA in a real world setting can reduce the burden of this disease.
